# Simple and complex retinal dystrophies are associated with profoundly different disease networks

**DOI:** 10.1038/srep41835

**Published:** 2017-01-31

**Authors:** Christina Kiel, Claire Lastrucci, Philip J. Luthert, Luis Serrano

**Affiliations:** 1 EMBL/CRG Systems Biology Research Unit, Centre for Genomic Regulation (CRG), Barcelona Institute of Science and Technology, Dr. Aiguader 88, 08003 Barcelona, Spain; 2 Universitat Pompeu Fabra (UPF), 08003 Barcelona, Spain; 3 Department of Ocular Biology and Therapeutics, UCL Institute of Ophthalmology, and NIHR Biomedical Research Centre, University College London, 11-43 Bath Street, London EC1V 9EL, UK; 4 Institució Catalana de Recerca i Estudis Avançats (ICREA), Pg. Lluís Companys 23, 08010 Barcelona, Spain

## Abstract

Retinopathies are a group of monogenetic or complex retinal diseases associated with high unmet medical need. Monogenic disorders are caused by rare genetic variation and usually arise early in life. Other diseases, such as age-related macular degeneration (AMD), develop late in life and are considered to be of complex origin as they develop from a combination of genetic, ageing, environmental and lifestyle risk factors. Here, we contrast the underlying disease networks and pathological mechanisms of monogenic as opposed to complex retinopathies, using AMD as an example of the latter. We show that, surprisingly, genes associated with the different forms of retinopathies in general do not overlap despite their overlapping retinal phenotypes. Further, AMD risk genes participate in multiple networks with interaction partners that link to different ubiquitous pathways affecting general tissue integrity and homeostasis. Thus AMD most likely represents an endophenotype with differing underlying pathogenesis in different subjects. Localising these pathomechanisms and processes within and across different retinal anatomical compartments provides a novel representation of AMD that may be extended to complex disease in general. This approach may generate improved treatment options that target multiple processes with the aim of restoring tissue homeostasis and maintaining vision.

Retinopathies, or retinal dystrophies (RD) are a group of diseases causing degeneration of a cell-rich layer in the eye, the retina^1^. Affected people generally experience a gradual loss of vision that may lead to blindness. Simple monogenic non-syndromic (M-NS) RD, such as retinitis pigmentosa (RP), congenital stationary night blindness, Leber’s congenital amaurosis, and rod-cone disease are caused by inherited rare germline mutations (Fig. 1a)^2^,^3^,^4^,^5^. The time of disease onset and progression is mainly driven by the genetic risk^6^ and the disease phenotype is restricted to the eye. Rare monogenic RD can also have a syndromic phenotype (M-S RD), predominantly cilia-associated, with multiple organs involved and pleiotropic effects, such as deafness, obesity, polydactyly, renal abnormalities, mild mental retardation, renal deficiency and metabolic disorders (e.g. in Usher and Bardet Biedl syndromes)^7^,^8^,^9^. In contrast, complex retinopathies (complex RD), such as age-related macular degeneration (AMD), have a later onset in life and are caused by a combination of age-related changes, common or rare genetic variations, and other risk factors such as lifestyle, smoking and gender^10^.

In recent years, the molecular basis of simple monogenic RD has expanded^11^,^12^,^13^,^14^,^15^,^16^,^17^,^18^,^19^. Experimental approaches to identify molecular components, their modulation, and arrangement into signalling cascades and interactomes have been successfully performed, and the connection to the physiological function in the retina and to the pathology has been established for most genes^20^,^21^,^22^,^23^,^24^,^25^. Likewise, for AMD, an example of a complex form of a retinopathy, pathogenic mechanisms have been established for certain risk genes^26^. For example, they participate in pathways with a known role in certain retinal compartments (e.g. retinal pigment epithelium (RPE) cells, Bruch’s membrane, extracellular matrix), such as the complement pathway and lipid metabolism^10^,^27^,^28^. Disease-causing proteins often work together, either in complexes or through participating in the same pathway. Therefore, network-based methods provide powerful approaches to achieve comprehensive understanding of the molecular basis underlying the disease and to investigate how the impact of genetic defects propagates across the network^29^,^30^. For example, biomarkers as readout of network perturbations can serve for disease classification and patient stratification. The identification of disease pathways may also illuminate new targets for drug development. Indeed, the knowledge of molecular disease mechanisms has been the basis for therapeutic approaches employing gene therapy in the eye^31^,^32^, retinal generation^33^, use of inhibitors^34^,^35^ or small molecules affecting metabolism^36^, and splice-switching oligonucleotides^37^,^38^.

In the present study, we contrast disease networks of genes associated with simple and complex forms of retinal dystrophies, using AMD as an example of the latter. By taking a network-centric approach, we show that the different types of retinal disorders are related to fundamentally different network properties. AMD proteins are associated with fragmented disease networks that link to multiple generic cell pathways. For many of them the physiological role with respect to vision- or ageing-related functions cannot be predicted based on the network analysis alone. We further observe differences with respect to the types of associated processes: while 66% of the genes implicated in monogenic and simple forms of the disease affect specific retinal cellular functions directly involved in vision (e.g. phototransduction, retinal recycling), only 7% of AMD risk genes are predicted to directly affect vision-related processes, but rather tend to affect tissue homeostasis processes (e.g. extracellular matrix remodelling, oxidative stress, inflammatory processes). Many of them are expected to play a role also in non-retinal tissues, consistent with their expression in many tissues, and possibly explaining the strong association of AMD genes to non-retinal syndromic phenotypes. Interestingly, some of the tissue homeostasis processes are also affected because of normal ageing-induced changes in tissue anatomy and physiology. We constructed an initial integrated network of processes within and across retinal compartments and their modulation through age-related changes in retinal anatomy and physiology, risk genes and their link to pathways, and via associations to environmental and lifestyle factors. This anatomical disease landscape for the first time demonstrates and visualizes the complexity of AMD pathogenesis. In future, the knowledge may guide improved and additional treatment options.

## Results

### Contrasting simple and complex retinopathies using a network-centric approach

**Figure 1:**
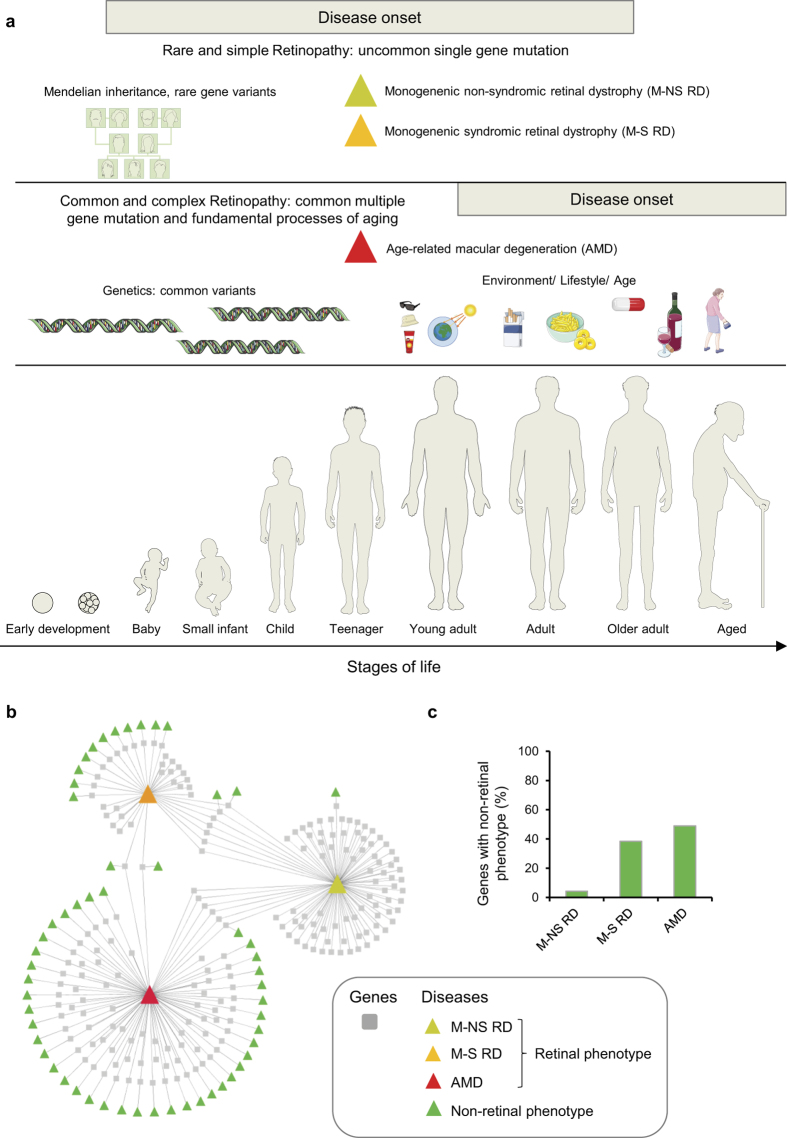
Principal differences between simple and complex retinal dystrophies (RD) and disease-gene network. (**a**) Monogenetic non-syndromic and syndromic RDs are mainly driven by rare genetic variation. The disease onset can be very early, if the infant is born blind or develops severe vision loss soon after birth. Vision loss can also develop later in life. Complex retinopathies, such as age-related macular degeneration (AMD) have a later onset in life and are caused by a combination of common genetic risk factors, ageing and environmental/lifestyle factors. Figure was prepared using images from Servier Medical Art by Servier (http://www.servier.com/Powerpoint-image-bank), which is licensed under a Creative Commons Attribution 3.0 Unported License. (**b**) Disease/phenotype-gene network of 208 genes related to RD. Boxes represent genes and triangles diseases or phenotypes. Monogenic non-syndromic RD are coloured in yellow, monogenic syndromic RD are coloured in orange, and AMD in red. Green triangles represent diseases or phenotypes that manifest in tissues outside the eye (non-vision related). (**c**) Percentage of genes with a non-retinal phenotype for M-NS RD, M-S RD, and AMD.

To contrast simple and complex retinopathies, we first used a classical gene- and network-centric molecular medicine approach, where the disease phenotype is considered as a consequence of perturbations of complex intra- and intercellular networks (e.g. disease-gene networks, protein-protein interaction networks, signalling/metabolic/and transcriptional networks; see Supplementary Fig. S1). Different genetic defects can result in similar disease phenotypes, indicating that diseases are interconnected (‘disease-gene network’ or ‘diseasome’)^29^,^39^. To establish the retinal diseasome we searched the literature and databases for genes linked to retinopathies and identified a total of 208 genes^1^,^10^,^27^,^28^,^40^ and the online mendelian inheritance in man (OMIM) database) (Supplementary Table S1). 96 genes are related to M-NS RD, 37 to M-S RD, and 90 genes are linked to AMD. For the union of 208 genes, the information for both retina-associated diseases and for all other (non-retinal phenotype) diseases was retrieved from the OMIM database and the respective disease-gene associations were depicted in a network (Fig. 1b). In this representation squared boxes represent genes and triangles represent diseases or phenotypes. The retinal diseasome discloses two remarkable features. First, there is a striking difference between the three retinal disease classes with respect to the number of genes that are additionally linked to a non-retinal phenotype. M-NS RDs, except for four genes, are not associated to a non-retinal tissue disease of the human body (Fig. 1c). As expected, a high number of non-retinal disease phenotypes are associated to genes causing M-S RD – likely because ciliary dysfunctions tend to affect multiple tissues and organs. The highest number of non-retinal associated phenotypes, however, is observed for AMD risk genes, affecting for example the kidney, skin, coronary arteries, and heart, or having neural or metabolic symptoms, or causing diabetes (see Supplementary Fig. S2). This substantiates that AMD is best considered a systemic disease. Second, the overlap of genes that are associated with both simple (M-NS and M-S) and complex retinopathies (AMD) is small. This suggests that the three disease classes influence distinct vision-related processes and functions in the retina.

**Figure 2:**
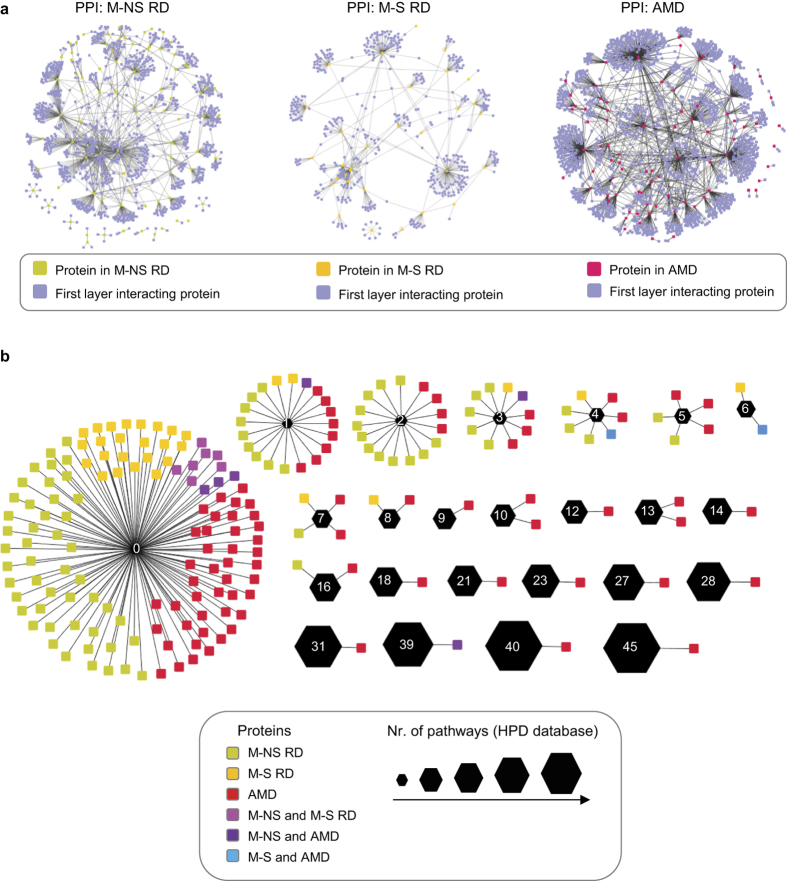
Protein interaction networks and pathways associated to retinal dystrophies. (**a**) Global protein interaction networks centred on RD gene products. Integrative literature based ‘omics’ protein-protein interaction networks for monogenic non-syndromic (M-NS), monogenic syndromic (M-S), and AMD-related disease gene products. The networks were retrieved from the HIPPIE database (http://cbdm.mdc-berlin.de/tools/hippie) and displayed using Cytoscape. (**b**) Association of proteins to different pathways based on the HPD database. Network representation of between the 208 RD proteins (round rectangles) and the number pathways a particular protein participates (black hexagons with white number inside indicating the number of pathways). The size of the hexagons correlates with the number of pathways a protein is associated to (see legend).

As genotype-phenotype relations often arise from a higher underlying complexity, the global analysis of interaction networks permits the understanding of the disease mechanism on a systems-network level^30^,^39^. We therefore asked if simple and complex retinal disorders are associated with different protein-protein interaction network topologies. To reconstruct networks among the risk genes, we used the HIPPIE integrated protein-protein interaction database (http://cbdm.mdc-berlin.de/tools/hippie), and retrieved interactions among the disease proteins and among the first layer of interacting proteins (Fig. 2a; Supplementary Table S2). For M-NS dystrophies the resulting network is well connected. In contrast, for complex retinopathies the derived network is fragmented with many AMD proteins acting as ‘hubs’ for a large number of interacting partners (high ‘degree’ for some nodes, see Supplementary Fig. S3). Accordingly, the AMD network has a 5.4-fold lower average clustering coefficient, indicating that proteins in the network link up with each other only to a limited degree (see Supplementary Fig. S4) and that they tend to have fewer shared neighbours (see Supplementary Fig. S5).

The high number of interaction partners for some nodes in the AMD network suggests that the same protein can link out to many different pathways. To analyse this, we extracted information from the HPD integrated database for human pathways^41^ (Supplementary Table S3). Many of the AMD proteins indeed participate in multiple signalling and metabolic pathways (Fig. 2b; see Supplementary Fig. S6). Further, many proteins belong to general cell signalling pathways (e.g. ERK, TGFb, TRAIL, STAT, JNK, HIF, PI3K), suggesting that they play a role in numerous processes, possibly also contributing to non-retinal tissue homeostasis. To support this hypothesis, we analysed the enrichment of proteins of the three RD classes for gene ontology (GO) processes using g:Profiler^42^,^43^ (see Supplementary Figs S7–S9). AMD genes are indeed not strongly enriched in GO processes (the average enrichment for GO processes follows the order of NS-RD > S-RD > AMD). Altogether, the AMD network and pathway properties corroborate the systemic and complex disease pathogenesis.

### Comparing processes affected in simple and complex retinal dystrophies

**Figure 3:**
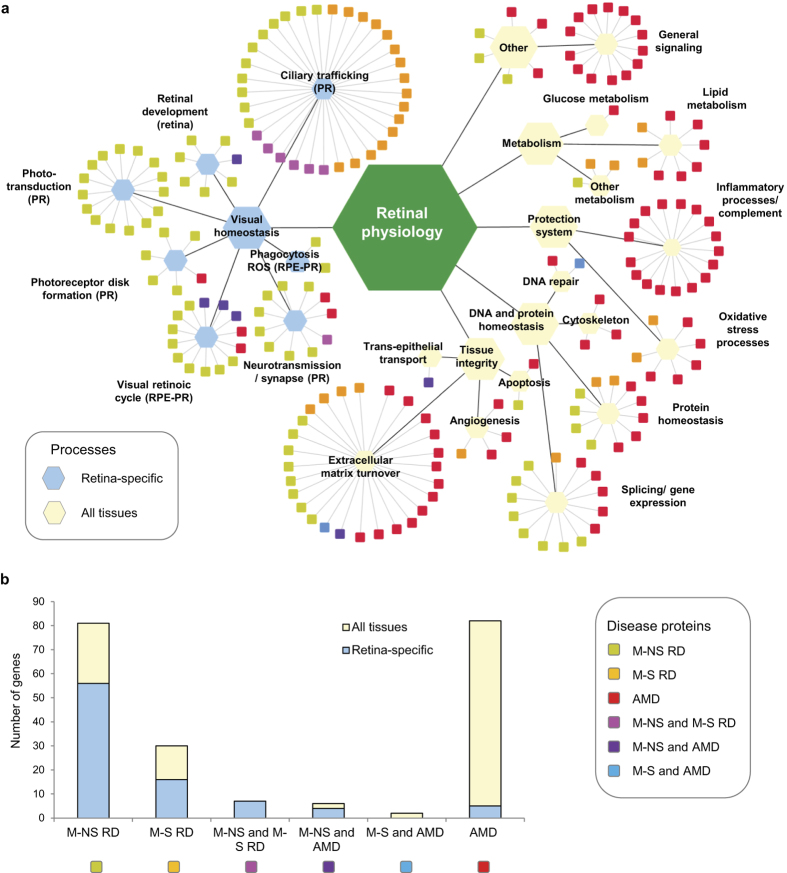
Functional classification of 208 RD genes and association to cell types or tissue compartments where this function contributes to vision-related tasks or to keeping the tissue integrity. (**a**) Boxes represent the 208 RD genes, which are coloured according to their RD disease classification. Genes are boxed according to their functional classification and assigned to either the retinal cell type where this process plays a role, or to functions affecting other retinal compartments and retinal tissue development and integrity, or to presumably cell general functions. (**b**) Statistic of proteins in the different disease classes and their association to retina-specific and cell and tissue general functions.

Based on the g:Profiler database and manual annotations using available literature, the 208 RD proteins were assigned to one (main) process and each process associated to a cell type or tissue compartment where this function is likely to play a role (‘protein-process networks’, Fig. 3a, Supplementary Table S1). In the resulting network representation, processes important for retinal physiology are organized hierarchically with six main classes. The first class is visual homeostasis with processes such as phototransduction, ciliary trafficking, neurotransmission, phagocytosis of outer segment disks, retinal development, and the visual retinal (Vitamin A) cycle. Indeed, most of the M-NS and M-S RD proteins link to these retina-specific processes (indicated as light blue hexagons in Fig. 3a). The remaining classes important for retinal physiology are tissue integrity (extracellular matrix turnover, angiogenesis, transepithelial transport, and apoptosis), DNA and protein homeostasis, the innate immune system (inflammatory and complement system processes), oxidative stress processes, metabolism, and a class with unknown proteins and those related to general signalling processes. These processes are expected to be important for retinal physiology, but at the same time they largely play a role in all (or most) human tissues (indicated as light yellow hexagons in Fig. 3a). The functional overlap of AMD-related genes with those of rare and simple RD is small and mainly concerns extracellular matrix turnover, splicing/gene expression and protein homeostasis. Overall, we observe that monogenic forms of RD are predominantly associated with retina-specific processes. Indeed, a few disease modules and pathways contain most of the disease-associated genes – supporting the validity of a network-centric approach. In contrast, AMD proteins are highly linked to processes that likely play a role in homeostasis in all tissues (Fig. 3b). The fact that AMD proteins are related to – presumably – general cell functions, such as general signalling, cytoskeleton, chaperones, Golgi function and apoptosis is in line with the fact that AMD genes are in general expressed at lower levels in both, neural retina and RPE/choroid^44^ (Supplementary Table S1; see Supplementary Fig. S10). However, some AMD genes are quite highly expressed in RPE/choroid^44^ (see Supplementary Fig. S10), which is consistent with the role of some AMD genes in the complement pathway, extracellular matrix remodelling, and lipid metabolism. However, many AMD gene products participate in general pathways and signalling modules that – presumably – control a multitude of cellular functions.

### An anatomy- and physiology-centric approach to understand complex and ageing-related retinopathies

**Figure 4:**
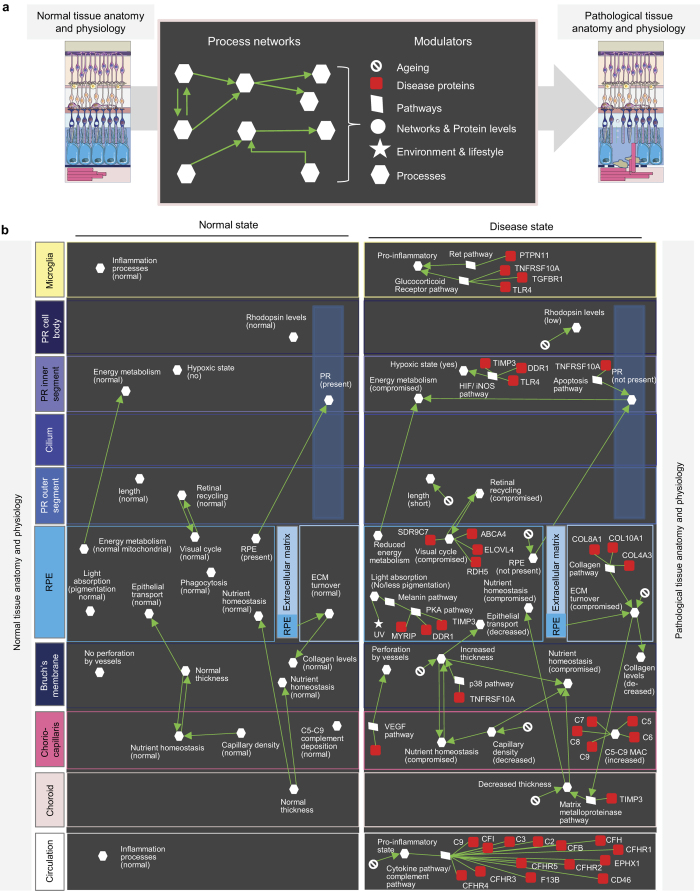
Anatomy- and physiology-centric approach to understand complex and ageing-related retinopathies and (patho)physiological process networks. (**a**) Schematic view summarizing the anatomy- and physiology-centric approach to molecular medicine where networks of processes are used to describe the anatomy and physiology, their interdependencies and modulation. (**b**) Selection of processes describing the tissue physiology in different retinal compartments and how they affect each other (see Supplementary Table S4 for a more complete list). On the left side the normal physiological process networks are shown and on the right side the pathological process networks together with their modulators (see symbols used in (**a**)).

There are two issues concerning the use of a classical molecular medicine approach to understand complex diseases such as AMD. First, as discussed above, many proteins associated to AMD are involved in ubiquitous pathways and it is not clear which networks, if any, are specifically important for vision-related functions. Second, a classical molecular medicine approach misses the contributions of normal ageing processes and the environment and lifestyle to the pathology. There are several examples of changes in retinal anatomy and physiology during normal ageing. For example, quantitative and linear changes have been reported for the choroidal capillary diameter (decreasing with age), Bruch’s membrane thickness (increases with age), and the capillary density (decrease with age)^45^ (Supplementary Table S4). All of this can affect the transport of nutrients, metabolites, waste products, etc. between compartments. Other ageing related changes affect the extracellular matrix turnover^46^ or the transition to a pro-inflammatory state during ageing^47^. Thus, in order to tackle complex diseases, a gene-/network- and pathway-centric (interactome) approach will not immediately take us to the physiologically relevant functions to allow understanding of disease and the identification of critical therapeutic targets. Likewise, simply focusing on the (patho)physiology provides only limited information for components and molecular networks underlying the pathology – especially if as in the case of AMD genes there only limited retina-specific expression. Therefore, we set out an alternative approach to understand complex and ageing-related diseases: an anatomy and physiology-centric molecular medicine approach (Fig. 4a). Here, we consider disease directly as a consequence of perturbations of tissue homeostasis. Tissue homeostasis in turn is best represented on the level of processes that can be modulated, e.g. by normal ageing or through disease associated proteins and pathways that link to those processes, but also through processes that affect each other within and across compartments (‘process networks’). We first performed a detailed literature analysis of all processes important in the different retinal compartments together with their interdependencies (Supplementary Table S4; see examples in Fig. 4b). We also indicated which processes are modulated through normal ageing and by environmental factors (e.g. UV light). Finally, a specific manual literature analysis was performed to find connections between processes and pathways (including cell general signalling pathways). If a link of a process to a pathway has been described previously in the literature, a connection to the respective disease protein was added via the respective pathway(s). Indeed, some of the cell general pathways could be linked to processes that have been shown previously to play a role for retinal functions (e.g. Bruchs’ membrane thickness and p38 pathway, melanin production in RPE cell and PKA pathway, collagen production in the extracellular matrix and the PI3K/Akt pathway).

The resulting (patho)physiological process map highlights many inter-dependencies, e.g. between the photoreceptor and the RPE (interdependencies of their energy metabolism) and between the RPE and Bruch’s membrane (epithelial transport of nutrients and waste products) (Fig. 4b). The process map also features that many dysregulated functions affect RPE cells, which thus stick out to be the critical cell type regulating retinal tissue cohesion. We also recognize innate immunity and inflammation processes turning up in several retinal compartments. Taken together, our AMD process network together with the different types of modulators represents an initial graphical representation of this disease, which can be refined over the coming years.

## Discussion

We have shown here that different forms of retinal dystrophies (monogenic or complex) are associated with fundamentally different disease networks. In particular, AMD risk genes participate in fragmented protein interaction networks with many interaction partners that are not connected. For those genes the associated cellular and physiological functions with respect to vision loss cannot be directly predicted based on the integration with published interaction networks. Further, it is likely that multiple pathways are affected, each through common genetic variation or general ageing processes that affect tissue physiology and predispose to disease manifestation at some point in life. Our integrated systems-level analysis shows that AMD pathogenesis likely manifests because of the deregulation of several cellular and tissue processes, suggesting that there are different forms of AMD with shared intermediate pathophenotypes (endophenotypes)^48^. We show that for complex diseases a gene-/network- and pathway-centric (interactome) approach does not guide the way to the physiologically distinct functions directly relevant to vision. To go beyond these limitations we propose to bridge information derived from physiology and interactomics and place tissue processes centre stage. In the future, integrating and connecting these processes within retinal cell and compartment states may also facilitate the generation of multiscale agent-based models^49^ of AMD pathogenesis. These models may serve as the cornerstone for integrative risk prediction models for patients.

The objective of classical molecular medicine is to understand the pathways and protein-protein interactions that link genes to changes in cellular functions and to elucidate how disease mutations affect protein activities, levels and network, with the final aim to identify targets for intervention. This network-centric view is probably biased by diseases such as cancer or monogenic diseases, which manifest rather on alterations of cellular processes, in the first place. The concept of protein-protein interactions, networks, and pathways is excellent for such ‘cellular diseases’, and enables therapeutic approaches that seek to inhibit protein interactions, inhibit protein activities, restore the folding of a protein, or express a non-functional protein through gene therapy. In order to tackle complex and ageing-related diseases such as AMD (similar concepts will also apply to arthritis, diabetes, and Alzheimer’s disease etc.), a pure cell network-centric approach may not be effective, as often tissue processes and tissue homeostasis is affected. First, because of the bias in studying networks in cells, we only have sparse information on networks across cells and tissues (exceptions are cell adhesion interactions^50^. Second, many of the ageing-related changes, rather than affecting interactions in tissues, affect the tissues physiology, integrity, and homeostasis. In our view, this known knowledge on pathological changes in retinal tissue homeostasis and processes should be the starting point to generate phenotypic descriptions of cells and compartments during AMD disease progression (processes and states). A critical task will be to find ways to integrate the physiology with ‘omics’ data – some approaches towards this goal are already taken within the ‘VPH/Physiome Project’ (http://www.vph-institute.org/; http://physiomeproject.org/), an effort towards a complete virtual description of human physiology that includes molecular details.

Understanding processes and tissue physiology may also have advantages compared to classical protein/network centric approaches and pave the way for improved and new therapeutic avenues. In fact, the beneficial effects of antioxidants already perfectly align with a process-based approach to therapy^51^,^52^,^53^. Exploring this further, other processes to be targeted for therapy could be tissue metabolism, i.e. affecting the interplay of lipid and glucose metabolic branches^54^. Likewise, targeting the complement system in retinal inflammatory diseases will require the comprehension of processes rather than individual protein-protein interactions^55^. In contrast, treatment of the later form of AMD using vascular endothelial growth factor (VEGF) inhibitors can be rather considered a classical gene/network-based therapy approach^56^. These inhibitors target a specific pathway responsible for stimulating growth of abnormal blood vessels in the retina. While they considerably slow down disease progression and may prevent vision loss for some time, they do not provide a real cure as retinal atrophy at later stages nevertheless occurs. Thus, treatments targeted towards tissue homeostasis may be an alternative and possibly superior approach compared to drugs that target directly interactions mediated by the disease gene – the common approach in current molecular medicine approaches taken for example in cancer. These interventions could be personalized by addressing one or more specific processes, depending on the patient’s genetic profile.

## Methods

### Disease-gene networks

The list of genes with established genetic associations to M-NS RD and M-S RD was based on databases (the online mendelian inheritance in man (OMIM) database, GeneReviews® (http://www.ncbi.nlm.nih.gov/books/NBK1417/)^40^, and a recent review article^1^. Genes associated to AMD were retrieved from the union of genes reported in several publication^10^,^27^,^28^,^57^ (Supplementary Table S5). Non-retinal phenotypes were extracted from the OMIM database and classified through manual literature searches according to the type of disease (Metabolism, Immune System, Diabetes, Developmental, Cancer) or the organ where they predominantly manifest (Coronary artery, Kidney, Blood system, Neuronal, Heart, Skin). The diseasome linking genes with phenotypes was represented using Cytoscape.

### Protein-protein interaction networks

The HIPPIE integrated protein-protein interaction database (http://cbdm.mdc-berlin.de/tools/hippie) was used to extract PPI information for risk gene products and their first layer of interacting proteins separately for the three disease classes (Supplementary Table S2). The networks were represented in cytoscape and the topological properties were analysed using the Cytoscape ‘Network Analysis’ plugin.

### Functional annotations

Functional annotations were retrieved through manual literature searches and using g:Profiler selecting ‘Biological Process’ as Gene Ontology (http://biit.cs.ut.ee/gprofiler/). If a gene is linked to more than one group it was assigned to the function/process that is more relevant to the known retinal biology function. For example, if a gene is linked to both cell cycle and cilia function, the ciliary function group was selected.

## Additional Information

**How to cite this article:** Kiel, C. *et al*. Simple and complex retinal dystrophies are associated with profoundly different disease networks. *Sci. Rep.* **7**, 41835; doi: 10.1038/srep41835 (2017).

**Publisher's note:** Springer Nature remains neutral with regard to jurisdictional claims in published maps and institutional affiliations.

## Supplementary Material

Supplementary Information

Supplementary Table S1

Supplementary Table S2

Supplementary Table S3

Supplementary Table S4

Supplementary Table S5
