# Supplementary material for: Simple and complex retinal dystrophies are associated with profoundly different disease networks
Source: Sci Rep. 2017 Jan 31;7:41835. doi: 10.1038/srep41835 (PMC5282568; doi:10.1038/srep41835)
Supplement: Supplementary Information [file srep41835-s1.pdf]

Supplementary information for:

# Simple and complex retinal dystrophies are associated with profoundly different disease networks

**Christina Kiel[1,2,*], Claire Lastrucci[1,2], Philip J. Luthert[3], Luis Serrano[1,2,4]**

[1]EMBL/CRG Systems Biology Research Unit, Centre for Genomic Regulation (CRG), Barcelona Institute of Science and Technology, Dr. Aiguader 88, 08003 Barcelona, Spain

[2]Universitat Pompeu Fabra (UPF), 08003 Barcelona, Spain

[3]Department of Ocular Biology and Therapeutics, UCL Institute of Ophthalmology, and NIHR Biomedical Research Centre, University College London, 11-43 Bath Street, London EC1V 9EL, UK

[4]Institució Catalana de Recerca i Estudis Avançats (ICREA), Pg. Lluís Companys 23, 08010 Barcelona, Spain

*christina.kiel@crg.eu

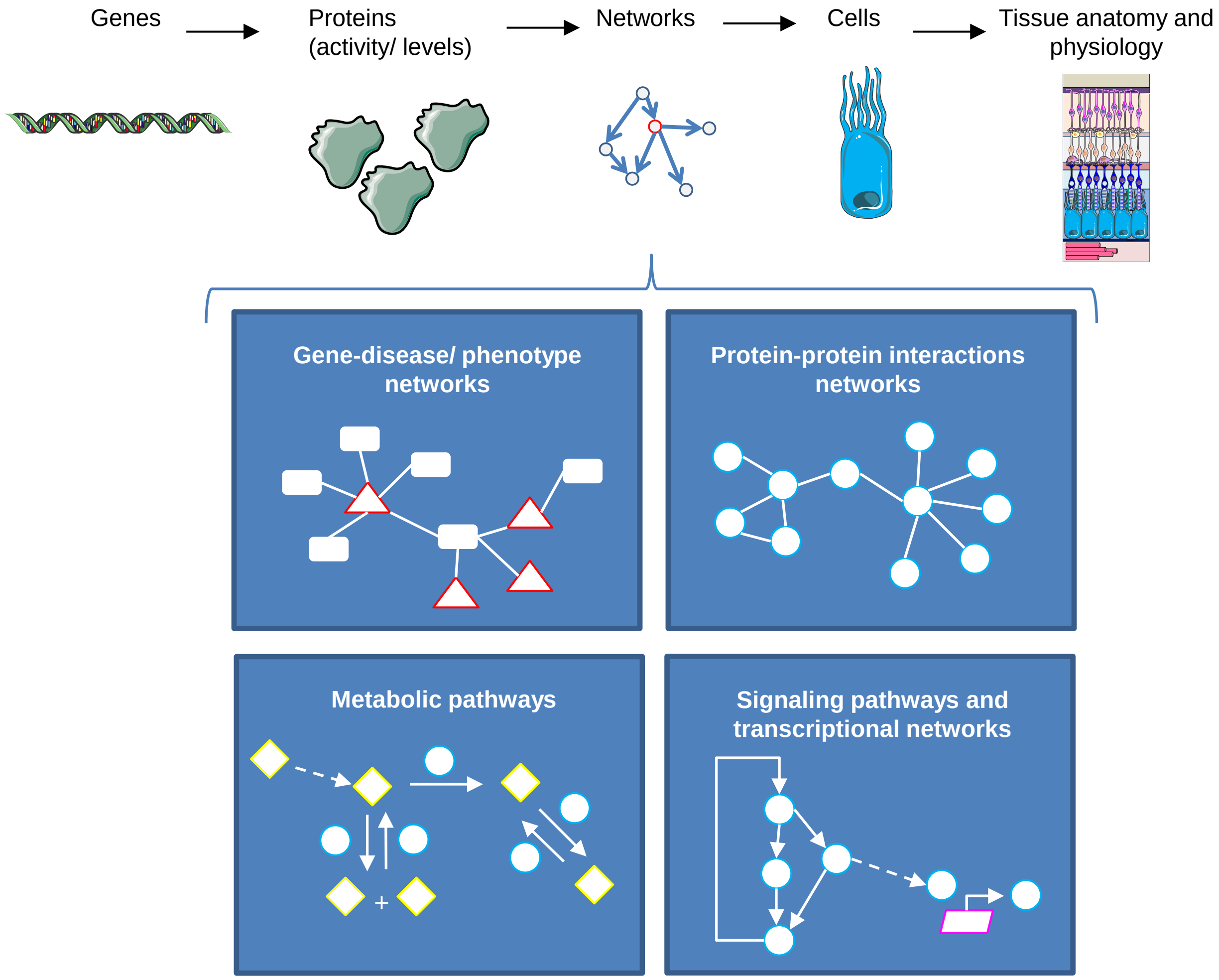


**Supplementary Figure 1**

**Schematic diagram illustrating a gene- and network-centric approach to molecular medicine.**

In this approach the disease phenotype is considered as the consequence of perturbations of complex intra- and intercellular networks caused by genetic defects that spread along the network, which ultimately affects the cellular or tissue phenotype. There are different types of networks to be considered. For example, in gene-disease networks genes participating in the same disease are linked. Protein-protein interaction networks refer the physical interactions between proteins. Metabolic, signalling and transcriptional pathways include directionality, activities and feedback mechanisms. The figure was prepared using images from Servier Medical Art by Servier (http://www.servier.com/Powerpoint-image-bank), which is licensed under a Creative Commons Attribution 3.0 Unported License.

Related to Figure 1b.

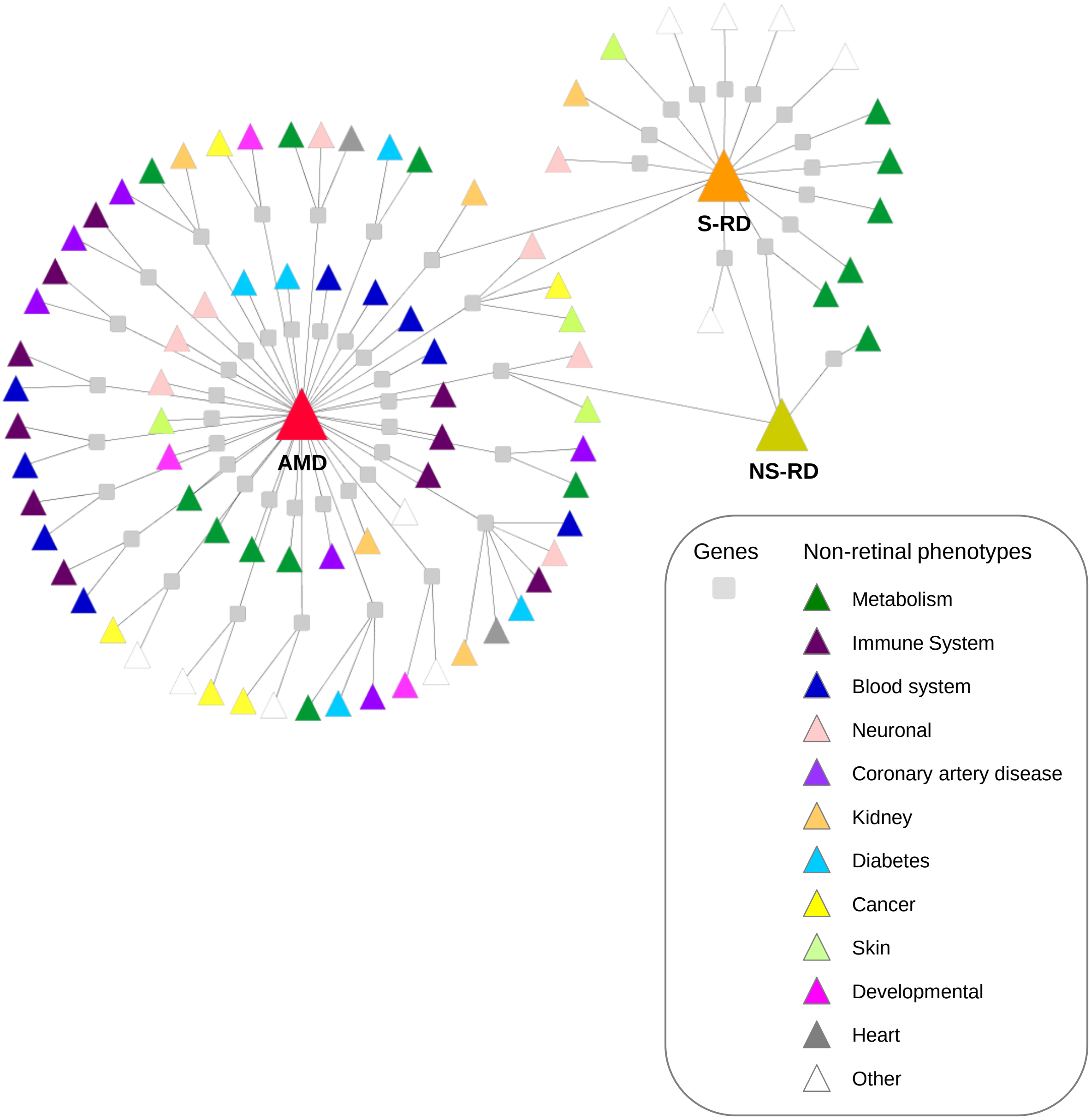


**Supplementary Figure 2**

**RD genes associated to non-retinal phenotypes.**

Network representation of the association between diseases and RD genes that additionally have a non-retinal phenotype. Boxes represent genes and triangles diseases or phenotypes. Monogenic non-syndromic RD are coloured in yellow, monogenic syndromic RD are coloured in orange, and AMD in red (see network). The remaining triangles represent disease or phenotypes that manifest in tissues outside the eye (non-vision related, see legend).

Related to Figure 1b.

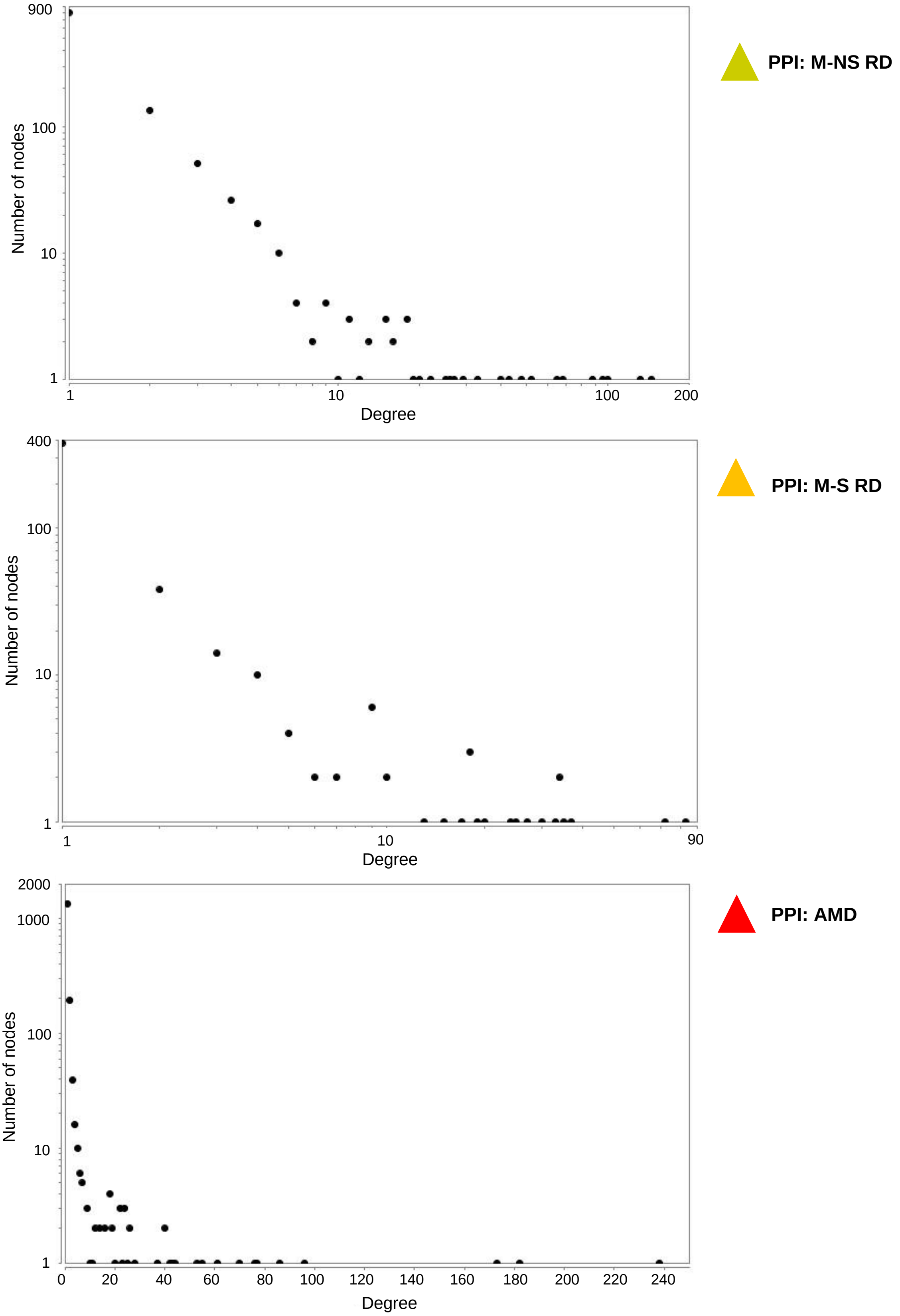


**Supplementary Figure 3**

**Degree distribution of proteins in the three protein-protein interaction (PPI) networks.**

Diagrams of the degree distributions (number of interaction partners) for all nodes (proteins) in the three PPI networks (M-NS RD, M-S RD, and AMD) are depicted. The network statistics and plots were calculated using the Cytoscape 'NetworkAnalyzer' plugin.

Related to Figure 2a.

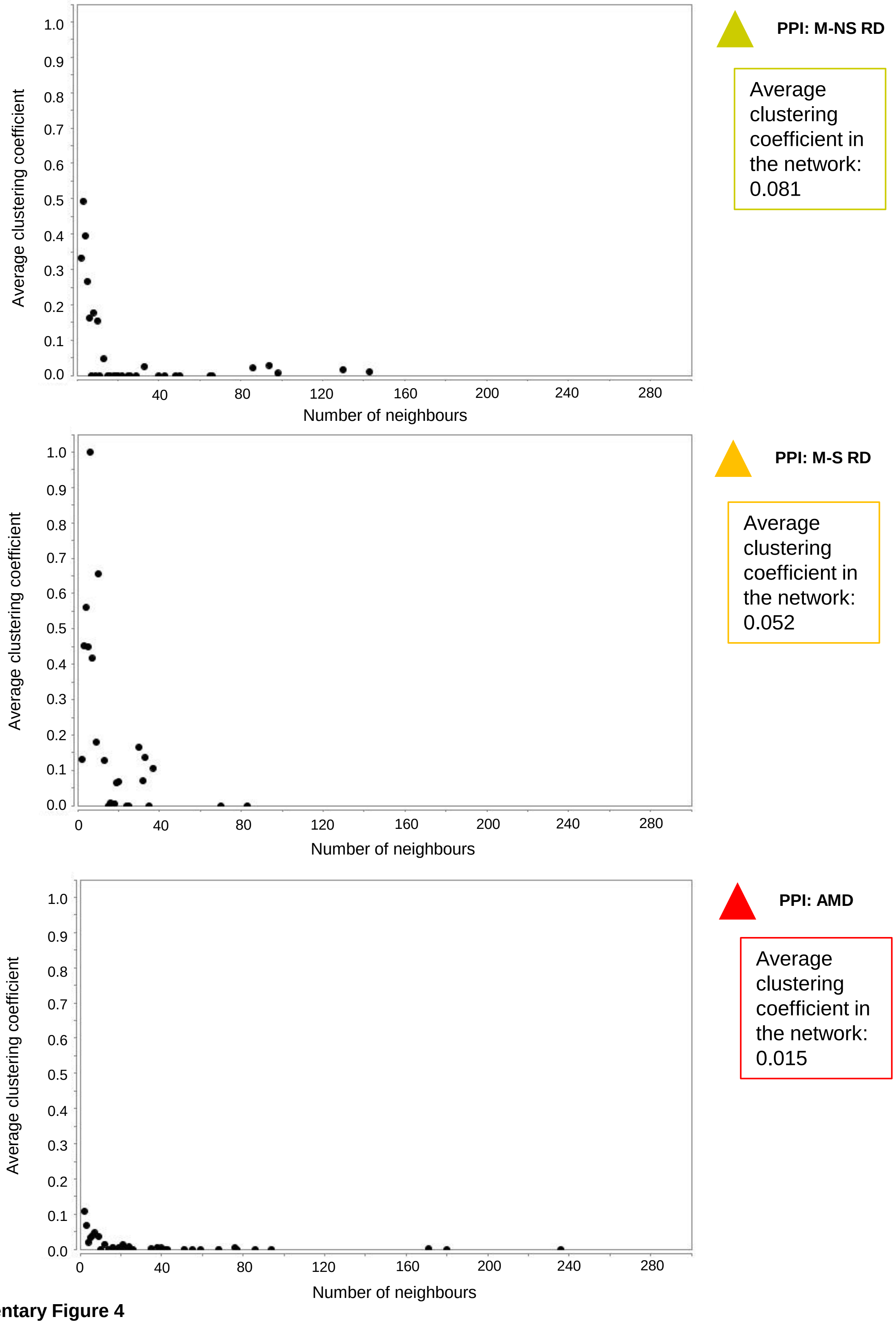


**Supplementary Figure 4**

**Average clustering distribution of proteins in the three protein-protein interaction (PPI) networks.**

Diagrams of the average clustering coefficients in the three PPI networks (M-NS RD, M-S RD, and AMD) are depicted. The network statistics and plots were calculated using the Cytoscape 'NetworkAnalyzer' plugin.

Related to Figure 2a.

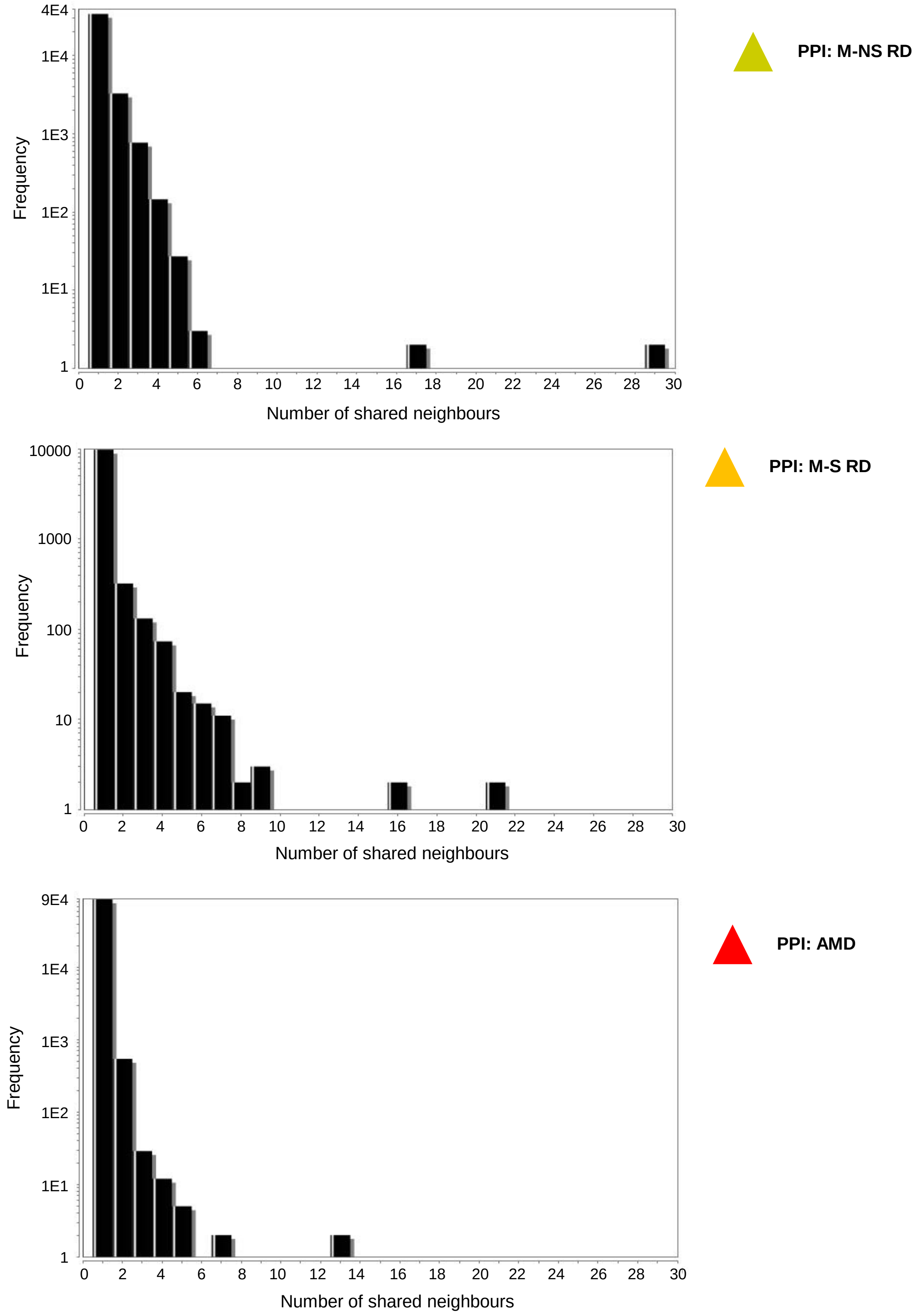


**Supplementary Figure 5**

**Shared neighbour distribution of proteins in the three protein-protein interaction (PPI) networks.**

Diagrams of the shared neighbour distribution in the three PPI networks (M-NS RD, M-S RD, and AMD) are depicted. The network statistics and plots were calculated using the Cytoscape 'NetworkAnalyzer' plugin.

Related to Figure 2a.

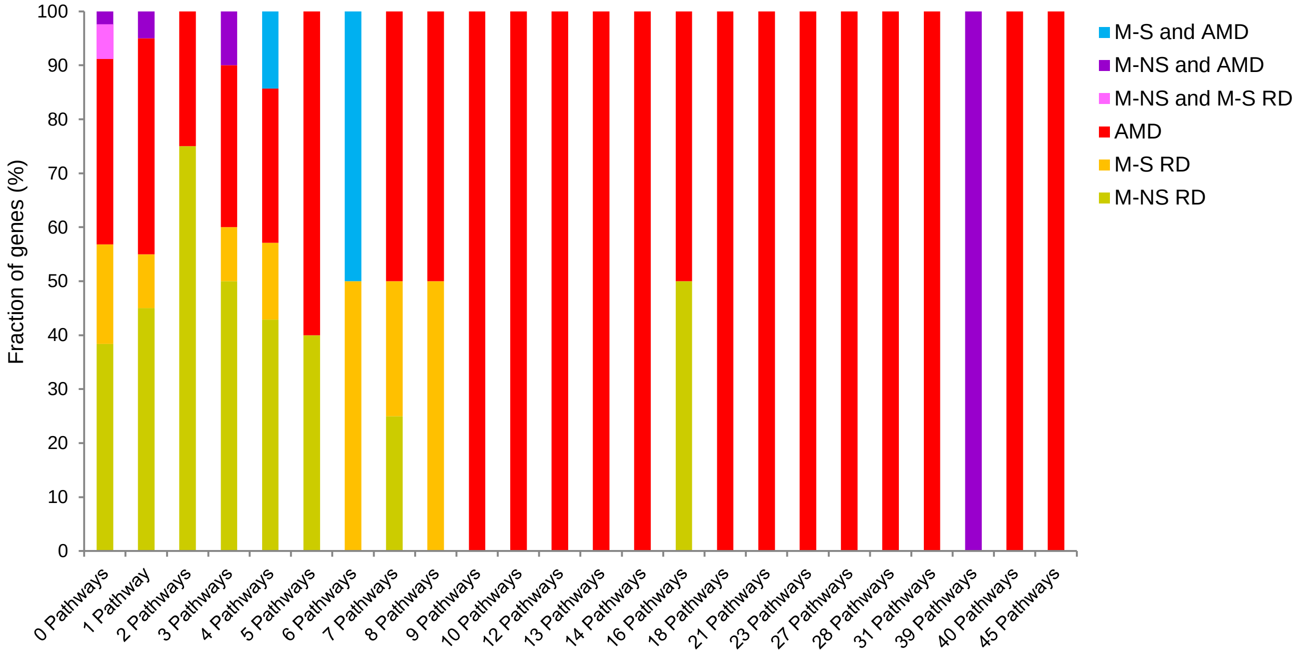


**Supplementary Figure 6**

**Fraction of proteins associated to different pathways based on the HPD database.**

Fraction of genes associated to a certain number of pathways coloured according to the different RD classes (see legend).

Related to Figure 2b.

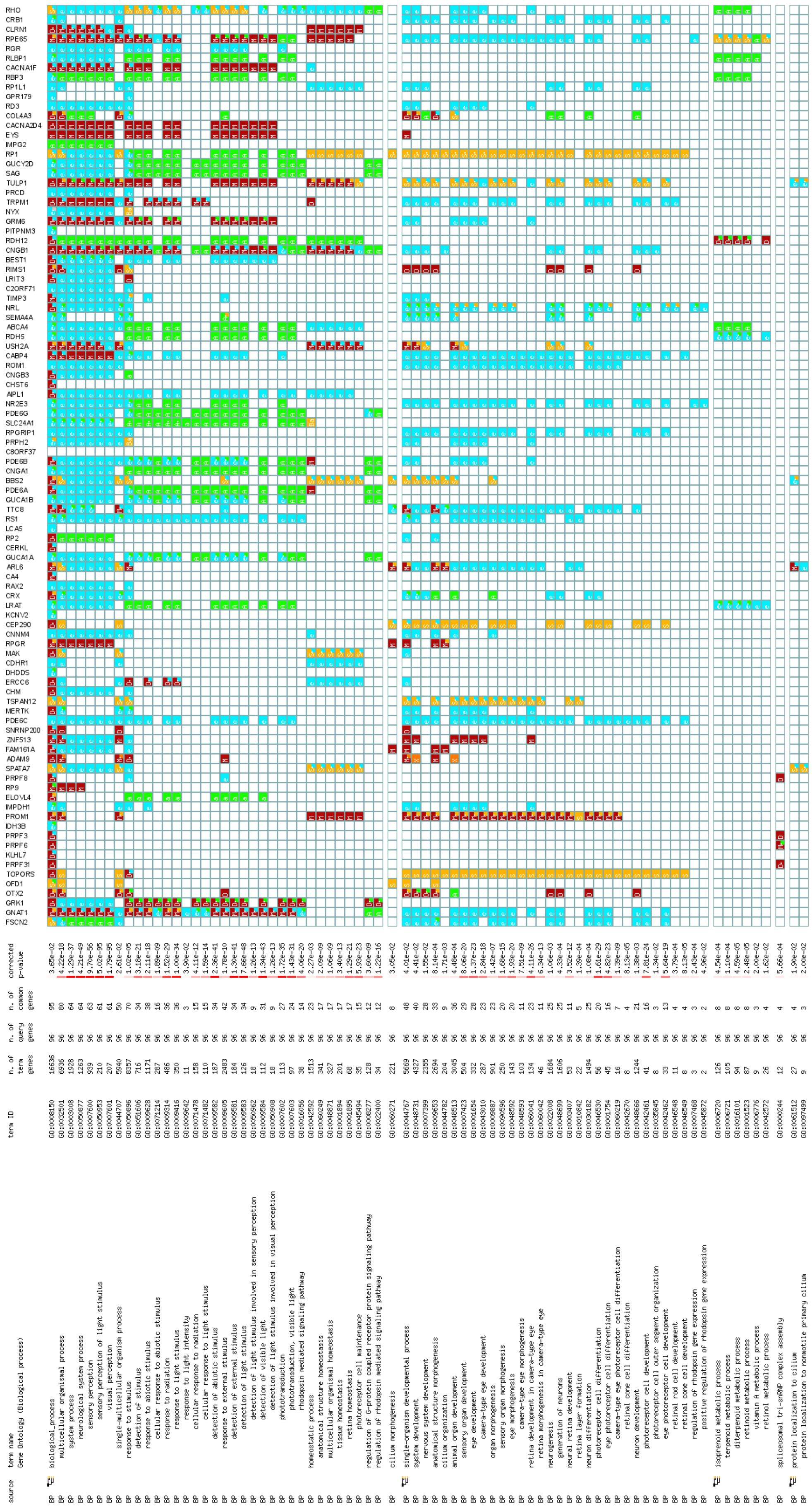


**Supplementary Figure 7**

**Supplementary Figure 7**

**Gene ontology process enrichment for M-NS RD.**

Enrichment analysis for the Gene ontology (GO) ‘Biological Process’ for M-NS RD genes using g:Profiler.

Related to Figure 3.

| source | | term name<br>Gene Ontology (Biological process) | term ID | n. of term genes | n. of query genes | n. of common genes | corrected p-value |
|---|---|---|---|---|---|---|---|
| BP | | protein localization to cilium | GO:0061512 | 27 | 35 | 4 | 3.96e-04 |
| BP | | protein localization | GO:0008104 | 2414 | 35 | 15 | 2.06e-02 |
| BP | | establishment of protein localization | GO:0045184 | 2011 | 35 | 14 | 1.28e-02 |
| BP | | single-organism process | GO:0044699 | 13451 | 35 | 35 | 1.01e-02 |
| BP | | single-organism cellular process | GO:0044763 | 12256 | 35 | 35 | 3.87e-04 |
| BP | | pigmentation | GO:0043473 | 93 | 35 | 7 | 7.55e-07 |
| BP | | cellular pigmentation | GO:0033059 | 49 | 35 | 7 | 7.25e-09 |
| BP | | developmental process | GO:0032502 | 5755 | 35 | 27 | 2.88e-05 |
| BP | | anatomical structure development | GO:0048856 | 5404 | 35 | 26 | 4.99e-05 |
| BP | | tissue development | GO:0009888 | 1707 | 35 | 13 | 1.20e-02 |
| BP | | epithelium development | GO:0060429 | 1051 | 35 | 11 | 3.93e-03 |
| BP | | single-organism developmental process | GO:0044767 | 5669 | 35 | 27 | 2.01e-05 |
| BP | | head development | GO:0060322 | 717 | 35 | 10 | 9.87e-04 |
| BP | | cellular developmental process | GO:0048869 | 4021 | 35 | 26 | 5.08e-08 |
| BP | | cell differentiation | GO:0030154 | 3731 | 35 | 21 | 3.77e-04 |
| BP | | fat cell differentiation | GO:0045444 | 198 | 35 | 8 | 5.20e-06 |
| BP | | cell development | GO:0048468 | 2149 | 35 | 15 | 4.78e-03 |
| BP | | anatomical structure morphogenesis | GO:0009653 | 2694 | 35 | 22 | 9.48e-08 |
| BP | | tissue morphogenesis | GO:0048729 | 575 | 35 | 10 | 1.27e-04 |
| BP | | morphogenesis of an epithelium | GO:0002009 | 442 | 35 | 8 | 2.55e-03 |
| BP | | epithelial tube morphogenesis | GO:0060562 | 308 | 35 | 6 | 4.48e-02 |
| BP | | anatomical structure formation involved in morphogenesis | GO:0048646 | 1120 | 35 | 15 | 7.70e-07 |
| BP | | multicellular organismal process | GO:0032501 | 6936 | 35 | 31 | 4.60e-07 |
| BP | | system process | GO:0003008 | 1928 | 35 | 21 | 1.46e-09 |
| BP | | neurological system process | GO:0050877 | 1263 | 35 | 21 | 3.16e-13 |
| BP | | neuromuscular process | GO:0050905 | 103 | 35 | 6 | 7.42e-05 |
| BP | | neuromuscular process controlling balance | GO:0050885 | 55 | 35 | 6 | 1.60e-06 |
| BP | | sensory perception | GO:0007600 | 939 | 35 | 21 | 7.49e-16 |
| BP | | sensory perception of light stimulus | GO:0050953 | 210 | 35 | 20 | 1.72e-27 |
| BP | | visual perception | GO:0007601 | 207 | 35 | 17 | 1.80e-21 |
| BP | | sensory perception of mechanical stimulus | GO:0050954 | 155 | 35 | 11 | 5.94e-12 |
| BP | | sensory perception of sound | GO:0007605 | 139 | 35 | 11 | 1.75e-12 |
| BP | | equilibrioception | GO:0050957 | 7 | 35 | 6 | 4.11e-13 |
| BP | | single-multicellular organism process | GO:0044707 | 5940 | 35 | 29 | 8.06e-07 |
| BP | | multicellular organism development | GO:0007275 | 4865 | 35 | 23 | 1.45e-03 |
| BP | | system development | GO:0048731 | 4327 | 35 | 22 | 9.20e-04 |
| BP | | nervous system development | GO:0007399 | 2355 | 35 | 21 | 7.14e-08 |
| BP | | neurogenesis | GO:0022008 | 1684 | 35 | 15 | 2.00e-04 |
| BP | | generation of neurons | GO:0048699 | 1606 | 35 | 15 | 1.06e-04 |
| BP | | neuron differentiation | GO:0030182 | 1494 | 35 | 14 | 3.48e-04 |
| BP | | photoreceptor cell differentiation | GO:0046530 | 56 | 35 | 5 | 1.34e-04 |
| BP | | mechanoreceptor differentiation | GO:0042490 | 61 | 35 | 6 | 3.04e-06 |
| BP | | neuron development | GO:0048666 | 1244 | 35 | 13 | 3.26e-04 |
| BP | | photoreceptor cell development | GO:0042461 | 41 | 35 | 4 | 2.24e-03 |
| BP | | animal organ development | GO:0048513 | 3045 | 35 | 19 | 4.75e-04 |
| BP | | sensory organ development | GO:0007423 | 504 | 35 | 13 | 5.34e-09 |
| BP | | ear development | GO:0043583 | 202 | 35 | 8 | 6.09e-06 |
| BP | | inner ear development | GO:0048839 | 176 | 35 | 8 | 2.05e-06 |
| BP | | inner ear receptor cell differentiation | GO:0060113 | 57 | 35 | 6 | 2.00e-06 |
| BP | | inner ear receptor cell development | GO:0060119 | 41 | 35 | 5 | 2.69e-05 |
| BP | | eye development | GO:0001654 | 332 | 35 | 8 | 2.91e-04 |
| BP | | organ morphogenesis | GO:0009887 | 901 | 35 | 14 | 5.34e-07 |
| BP | | sensory organ morphogenesis | GO:0090596 | 250 | 35 | 8 | 3.24e-05 |
| BP | | eye morphogenesis | GO:0048592 | 143 | 35 | 6 | 5.26e-04 |
| BP | | eye photoreceptor cell differentiation | GO:0001754 | 45 | 35 | 4 | 3.29e-03 |
| BP | | brain development | GO:0007420 | 678 | 35 | 9 | 6.34e-03 |
| BP | | embryo development | GO:0009790 | 947 | 35 | 10 | 1.22e-02 |
| BP | | embryonic morphogenesis | GO:0048598 | 568 | 35 | 10 | 1.13e-04 |
| BP | | embryonic organ development | GO:0048568 | 413 | 35 | 10 | 5.47e-06 |
| BP | | embryonic organ morphogenesis | GO:0048562 | 286 | 35 | 9 | 4.05e-06 |
| BP | | embryonic heart tube development | GO:0035050 | 75 | 35 | 5 | 5.92e-04 |
| BP | | embryonic heart tube morphogenesis | GO:0003143 | 65 | 35 | 5 | 2.87e-04 |
| BP | | pattern specification process | GO:0007389 | 438 | 35 | 9 | 1.63e-04 |
| BP | | specification of symmetry | GO:0009799 | 123 | 35 | 8 | 1.16e-07 |
| BP | | determination of bilateral symmetry | GO:0009855 | 122 | 35 | 8 | 1.09e-07 |
| BP | | determination of left/right symmetry | GO:0007368 | 114 | 35 | 8 | 6.27e-08 |
| BP | | determination of heart left/right asymmetry | GO:0061371 | 63 | 35 | 5 | 2.45e-04 |
| BP | | heart looping | GO:0001947 | 60 | 35 | 5 | 1.91e-04 |
| BP | | cellular component organization or biogenesis | GO:0071840 | 6111 | 35 | 29 | 1.71e-06 |
| BP | | cellular component biogenesis | GO:0044085 | 2545 | 35 | 18 | 1.83e-04 |
| BP | | homeostatic process | GO:0042592 | 1513 | 35 | 16 | 5.01e-06 |
| BP | | anatomical structure homeostasis | GO:0060249 | 341 | 35 | 16 | 5.27e-16 |
| BP | | multicellular organismal homeostasis | GO:0048871 | 327 | 35 | 16 | 2.69e-16 |
| BP | | tissue homeostasis | GO:0001894 | 201 | 35 | 16 | 9.89e-20 |
| BP | | retina homeostasis | GO:0001895 | 68 | 35 | 14 | 6.53e-23 |
| BP | | photoreceptor cell maintenance | GO:0045494 | 35 | 35 | 13 | 7.03e-25 |
| BP | | organelle localization | GO:0051640 | 444 | 35 | 8 | 2.64e-03 |
| BP | | vesicle localization | GO:0051648 | 230 | 35 | 7 | 4.12e-04 |
| BP | | pigment granule localization | GO:0051875 | 26 | 35 | 7 | 5.72e-11 |
| BP | | melanosome localization | GO:0032400 | 25 | 35 | 6 | 1.02e-08 |
| BP | | cellular component organization | GO:0016043 | 5985 | 35 | 29 | 9.85e-07 |
| BP | | cellular component morphogenesis | GO:0032989 | 1517 | 35 | 18 | 4.12e-08 |
| BP | | cell part morphogenesis | GO:0032990 | 1109 | 35 | 17 | 3.39e-09 |
| BP | | cell morphogenesis | GO:0000902 | 1444 | 35 | 18 | 1.80e-08 |
| BP | | organelle organization | GO:0006996 | 3556 | 35 | 24 | 3.16e-07 |
| BP | | single-organism organelle organization | GO:1902589 | 2543 | 35 | 20 | 2.90e-06 |
| BP | | cellular component assembly | GO:0022607 | 2353 | 35 | 18 | 5.33e-05 |
| BP | | cellular component assembly involved in morphogenesis | GO:0010927 | 273 | 35 | 14 | 4.47e-14 |
| BP | | organelle assembly | GO:0070925 | 546 | 35 | 15 | 2.61e-11 |
| BP | | cell projection organization | GO:0030030 | 1488 | 35 | 24 | 8.65e-16 |
| BP | | cilium organization | GO:0044782 | 204 | 35 | 15 | 1.01e-17 |
| BP | | cell projection assembly | GO:0030031 | 359 | 35 | 15 | 5.27e-14 |
| BP | | cell projection morphogenesis | GO:0048858 | 1090 | 35 | 17 | 2.57e-09 |
| BP | | cilium morphogenesis | GO:0060271 | 221 | 35 | 15 | 3.45e-17 |
| BP | | cilium assembly | GO:0042384 | 187 | 35 | 14 | 2.09e-16 |
| BP | | nonmotile primary cilium assembly | GO:0035058 | 24 | 35 | 8 | 9.38e-14 |
| BP | | inner ear receptor stereocilium organization | GO:0060122 | 26 | 35 | 5 | 2.41e-06 |
| BP | | establishment of organelle localization | GO:0051656 | 385 | 35 | 7 | 1.29e-02 |
| BP | | sensory processing | GO:0050893 | 3 | 35 | 2 | 2.42e-02 |
| BP | | establishment of vesicle localization | GO:0051650 | 219 | 35 | 7 | 2.95e-04 |
| BP | | establishment of pigment granule localization | GO:0051905 | 24 | 35 | 7 | 3.01e-11 |
| BP | | pigment granule aggregation in cell center | GO:0051877 | 2 | 35 | 2 | 8.09e-03 |
| BP | | establishment of melanosome localization | GO:0032401 | 23 | 35 | 6 | 5.81e-09 |
| BP | | multicellular organism growth | GO:0035264 | 145 | 35 | 5 | 1.56e-02 |
| BP | | subpallium development | GO:0021544 | 22 | 35 | 3 | 2.09e-02 |
| BP | | striatum development | GO:0021756 | 16 | 35 | 3 | 7.67e-03 |
| BP | | pigment granule transport | GO:0051904 | 23 | 35 | 7 | 2.14e-11 |
| BP | | melanosome transport | GO:0032402 | 22 | 35 | 6 | 4.30e-09 |
| BP | | cilium movement | GO:0003341 | 43 | 35 | 4 | 2.73e-03 |
| BP | | cilium or flagellum-dependent cell motility | GO:0001539 | 21 | 35 | 3 | 1.81e-02 |
| BP | | cilium-dependent cell motility | GO:0060285 | 15 | 35 | 3 | 6.24e-03 |
| BP | | regulation of microtubule-based movement | GO:0060632 | 19 | 35 | 3 | 1.32e-02 |
| BP | | regulation of cilium movement | GO:0003352 | 12 | 35 | 3 | 3.03e-03 |
| BP | | regulation of cilium-dependent cell motility | GO:1902019 | 6 | 35 | 3 | 2.78e-04 |
| BP | | regulation of cilium beat frequency | GO:0003356 | 11 | 35 | 3 | 2.28e-03 |
| BP | | cilium movement involved in cell motility | GO:0060294 | 11 | 35 | 3 | 2.28e-03 |
| BP | | regulation of cilium movement involved in cell motility | GO:0060295 | 6 | 35 | 3 | 2.78e-04 |
| BP | | regulation of cilium beat frequency involved in ciliary motility | GO:0060296 | 6 | 35 | 3 | 2.78e-04 |
| BP | | chaperone-mediated protein complex assembly | GO:0051131 | 15 | 35 | 3 | 6.24e-03 |
| BP | | response to leptin | GO:0044321 | 20 | 35 | 3 | 1.55e-02 |
| BP | | cellular response to leptin stimulus | GO:0044320 | 16 | 35 | 3 | 7.67e-03 |
| BP | | regulation of appetite | GO:0032098 | 24 | 35 | 3 | 2.75e-02 |
| BP | | regulation of response to food | GO:0032095 | 20 | 35 | 3 | 1.55e-02 |
| BP | | negative regulation of response to food | GO:0032096 | 13 | 35 | 3 | 3.93e-03 |
| BP | | negative regulation of appetite | GO:0032099 | 13 | 35 | 3 | 3.93e-03 |
| BP | | leptin-mediated signaling pathway | GO:0033210 | 9 | 35 | 3 | 1.16e-03 |
| BP | | negative regulation of appetite by leptin-mediated signaling pathway | GO:0038108 | 4 | 35 | 3 | 5.56e-05 |

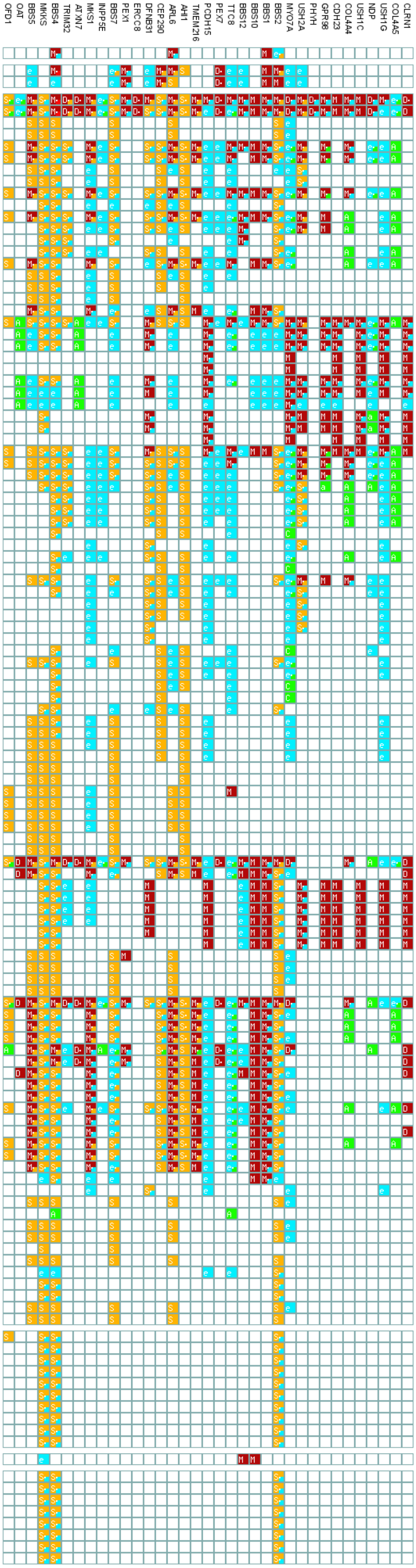


**Supplementary Figure 8**

**Supplementary Figure 8**

**Gene ontology process enrichment for M-S RD.**

Enrichment analysis for the Gene ontology (GO) ‘Biological Process’ for M-S RD genes using g:Profiler.

Related to Figure 3.

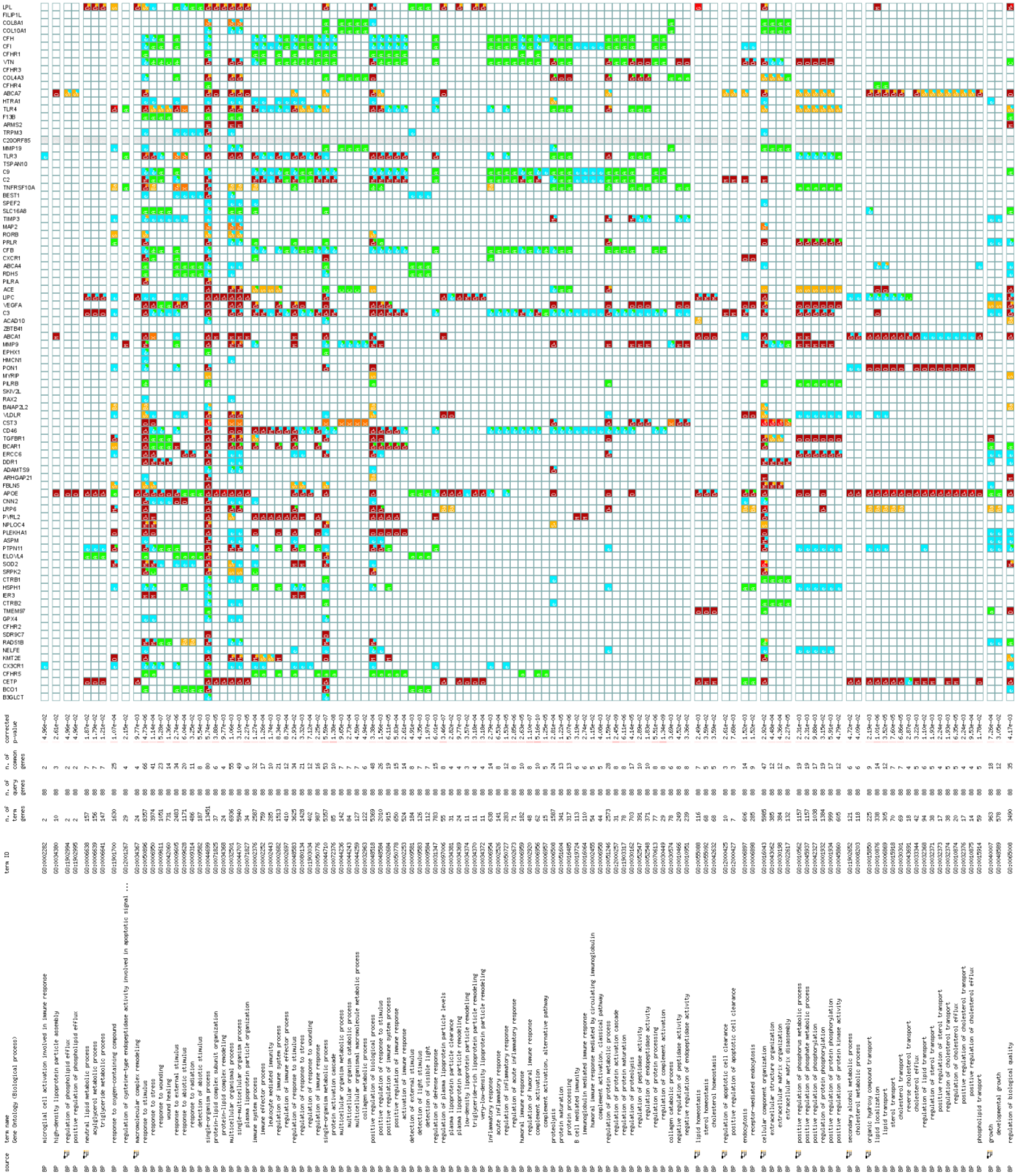

**Supplementary Figure 9**

**Gene ontology process enrichment for AMD.**

Enrichment analysis for the Gene ontology (GO) ‘Biological Process’ for AMD genes using g:Profiler.

Related to Figure 3.

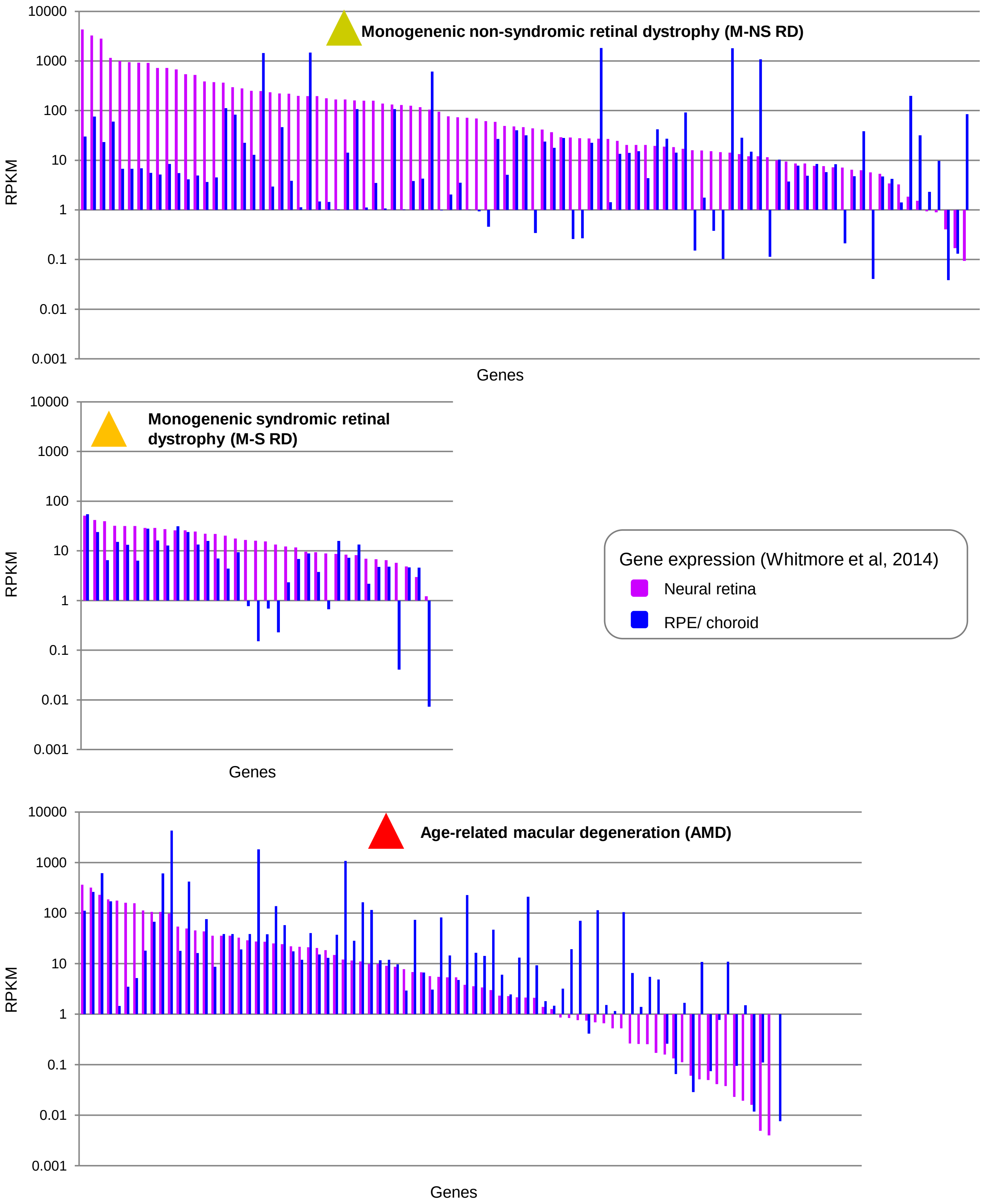


**Supplementary Figure 10**

**Gene expression levels in retinal tissues.**

Average gene expression levels in neural retina and RPE/ choroid (see Whitmore et al, 2014) displayed separately for genes related to monogenic non-syndromic RD, monogenic syndromic RD, and AMD. Genes are sorted by decreasing expression levels in neural retina.

Related to Figure 4.

## Supplementary Tables (online)

### Supplementary Table S1

**Disease associations of 208 genes related to retinal dystrophies (RD), functional classification and expression levels in retinal cell types.**

Related to Figure1b, Figure 3 and Figure 4.

### Supplementary Table S2

**Interaction networks reconstructed among RD genes using the HIPPIE database.**

Related to Figure 2a.

### Supplementary Table S3

**Pathway information obtained from the HPD database.**

Related to Figure 2b.

### Supplementary Table S4

**Processes and modulators in the physiological condition, during ageing, and in AMD pathogenesis.**

Related to Figure 4.

### Supplementary Table S5

**Generation of a list of genes associated to AMD.**

Related to Figure 1b.
